# Supplementary material for: The Effects of ESC/ESH-Based Written Generic Lifestyle Advice and a Nutraceutical on 24-Hour Blood Pressure in Patients with High–Normal Office Blood Pressure and Low–Moderate Cardiovascular Risk
Source: Nutrients. 2023 Dec 13;15(24):5099. doi: 10.3390/nu15245099 (PMC10745575; doi:10.3390/nu15245099)
Supplement: Supplementary file 1 [file nutrients-15-05099-s001.zip › nutrients-2754361-supplementary.pdf]

Little steps toward a healthy lifestyle

Behavioural and dietary advice derived from the 2018 ESC/ESH Guidelines for the management of arterial hypertension and adapted for better patient education by the ESH Excellence Hypertension Centre, IRCCS INRCA, Ancona, Italy.

High blood pressure is the leading cause of the most common cardiovascular diseases (stroke, myocardial infarction, heart failure, kidney failure, dementia) and related mortality.

With simple steps, you can achieve important results by reducing your arterial blood pressure, which is already harmful at values defined as high-normal (≥130/85 mmHg).

How to avoid ending up inside the cage of this silent killer?

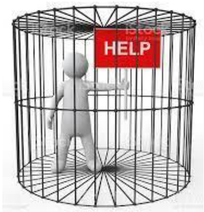

And once inside, how to get out?

Here are some simple and practical tips:

1. LIMIT SALT ASSUMPTION:
Daily intake of no more than 5 grams of sodium is recommended.
Avoid or limit foods such as cured meats, cold cuts, cheese (especially aged), pizza, canned foods and "junk food" such as chips, french fries, fast food, and appetizer snacks.
Always prefer potassium-rich foods over sodium-rich ones and possibly use salt substitutes made with potassium salts.

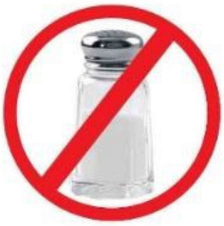

2. MODERATE ALCOHOL CONSUMPTION:
Drink occasionally!!!
Excessive alcohol consumption increases blood pressure.
MEN: < of 14 units of alcohol per week
WOMEN: < of 8 units of alcohol per week
(1 unit of alcohol = 125 ml of wine, 250 ml of beer, or one spirit shot).

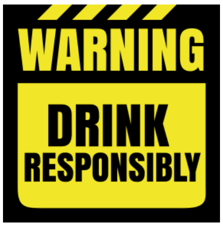

3. CHECK YOUR DIET and REACH YOUR SHAPE WEIGHT:
Increase your consumption of vegetables (≥2-3 servings/day), fresh fruits (≥2-3 servings/day), fish (1-2 times a week), dried fruits (e.g., 30 g/day) and unsaturated fatty acids (olive oil).
Limit intake of red meat (maximum 350 to 500 g/week) and unsaturated fatty acids (butter, lard, cream, etc.).
Avoid sugary foods or drinks if possible.

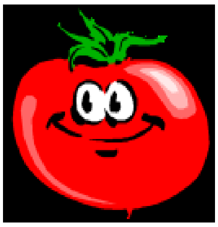

4. DO PHYSICAL ACTIVITY:
Dedicate, consistently, at least 30 minutes a day for a fast walk on level ground for 5-7 days a week or do similar physical activities daily.
Increase movement by all means (i.e. don't take elevators but the stairs).

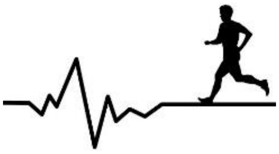

5. STOP SMOKING:
Smoking would nullify all the efforts made. Why spend to get sick?
In addition to heart and brain diseases, smoking causes all kinds of cancers.

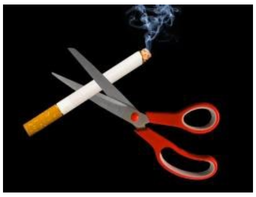

IT ALL DEPENDS ON YOU!!! PLEASE DON'T LET YOURSELF DOWN!!!
